# Older Adults Living With Food Insecurity: The Impact on Psychological Health

**DOI:** 10.1093/geroni/igaa057.1218

**Published:** 2020-12-16

**Authors:** Robina Sandhu, Victoria Marshall, Carolyn Becker, Lisa Kilpela, Keesha Middlemass

**Affiliations:** 1 UT Health San Antonio, San Antonio, Texas, United States; 2 UT Health San Antonio, San Antonio, United States; 3 Trinity University, San Antonio, Texas, United States; 4 Howard University, Washington D.C., District of Columbia, United States

## Abstract

Food insecurity (FI) refers to inadequate access to nutritious foods, either in terms of quality or quantity. In older adults, FI is associated with functional impairment, isolation, financial vulnerability, lower quality of life, and poorer health (e.g., diabetes, cardiovascular disease; Fernandes, et al). The effects of FI on psychological health broadly, however, have not been well-documented among older adults. This study sought to examine the impact of FI severity on psychological health indices among older adults. Older adult clients of local food pantries completed self-report measures of FI severity, worry, internalized weight stigma, trauma history, and eating disorder (ED) symptoms/behaviors. Participants (N=124, aged 66+) included: 68.5% women, 67.7% Hispanic, 75.8% □high school education, 51.0% household income < $10,000/year. Controlling for gender in all analyses, results indicated that FI severity predicted increased worry (p < .001, 21.9% variance), greater internalized weight stigma (p = .04, 3.9% variance), and a trend for increased risk for lifetime traumatic event exposure (OR = 1.4, 95% CI [.98, 2.01]). Regarding ED symptoms, male gender (OR = 6.60, 95% CI [1.96, 22.23] and higher FI severity predicted risk for self-induced vomiting in the past month (OR = 2.5, 95% CI [1.15, 5.36], risk for laxative/diuretic use for weight control (OR = 2.16, 95% CI [1.03, 4.52], and greater dietary restraint (p < .001, 16.1% variance). Male gender was associated with higher risk for binge eating in the past month (OR = 3.19, 95% CI [1.10, 9.24], while FI severity was not. Implications will be discussed.

